# Draft Genome Sequence of *Lactobacillus sucicola* JCM 15457^T^, a Motile Lactic Acid Bacterium Isolated from Oak Sap

**DOI:** 10.1128/genomeA.00403-14

**Published:** 2014-05-08

**Authors:** Tomohiro Irisawa, Kenshiro Oshima, Wataru Suda, Maki Kitahara, Mitsuo Sakamoto, Keiko Kitamura, Toshiya Iida, Masahiro Hattori, Moriya Ohkuma

**Affiliations:** a Japan Collection of Microorganisms/Microbe Division, RIKEN BioResource Center, Tsukuba, Ibaraki, Japan; b Center for Omics and Bioinformatics, Graduate School of Frontier Sciences, the University of Tokyo, Kashiwa, Chiba, Japan

## Abstract

Here, we report the draft genome sequence of a motile lactic acid bacterium, *Lactobacillus sucicola* JCM 15457^T^, isolated from oak sap. Motility-related genes and their organization in the annotated genome were broadly similar to those in the sequenced genomes of related lactobacilli.

## GENOME ANNOUNCEMENT

Lactic acid bacteria in the genus *Lactobacillus* occur in various environments such as plants, sewage, foods (dairy products, fermented meat, sourdough, vegetables, fruits, and beverages), and respiratory, gastrointestinal, and other tracts of animals, including human and insects. Although *Lactobacillus* members have been generally known as nonmotile, it has been recognized in recent years that some species affiliated with the *Lactobacillus salivarius* phylogenetic clade show motile ability (1). Motility-related genes have been identified in the sequenced genomes of strains in the *L. salivarius* clade; these strains are human and cow gastrointestinal isolates of *Lactobacillus ruminis* and a wine must/cider isolate of *Lactobacillus mali* (1). *Lactobacillus sucicola* strain JCM 15457^T^, isolated from the sap of an oak tree (*Quercus* sp.), is also affiliated with the *L. salivarius* clade and shows motility (2); therefore, its genome sequencing and comparative studies will be beneficial to our understanding of the nature of motility in this genus.

Whole-genome sequencing of *L. sucicola* JCM 15457^T^ was performed using an Ion Torrent PGM system. A total of 468,556 high-quality reads were assembled by Newbler v. 2.8 (454 Life Sciences) into 15 contigs with an *N*_50_ length of 402,418 bp. The resulting draft genome sequence was 2,454,642 bp, with 42.6× redundancy and a G+C content of 38.4%. The draft genome sequence was annotated by the RAST server (3) using Glimmer3 (4). The *L. sucicola* JCM 15457^T^ genome contained 2,409 protein-coding sequences and 3 rRNA and 49 tRNA coding sequences.

In our preliminary analyses of the genome sequence of *L. sucicola* JCM 15457^T^, 44 genes involving flagellum biogenesis and chemotaxis were identified. Their gene organization is broadly similar to those in the sequenced genomes of *L. ruminis* and *L. mali*. *L. sucicola* JCM 15457^T^ was found to carry only one flagellin gene (*fliC*) (as in *L. ruminis* ATCC 25644 [human isolate] and *L. mali* DSM 20444^T^), whereas *L. ruminis* ATCC 27782 (cow isolate) carries a second gene (*fliC2*) as an abundantly expressed flagellin gene (1). The genome information for *L. sucicola* JCM 15457^T^ will be useful for studies of the nature of motility in lactobacilli and its relation to their ecology.

### Nucleotide sequence accession numbers.

The draft genome sequence of *L. sucicola* JCM 15457^T^ has been deposited in DDBJ/EMBL/GenBank under the accession numbers BALC01000001 through BALC01000015.
